# Edmonton Obesity Staging System Prevalence and Association with Weight Loss in a Publicly Funded Referral-Based Obesity Clinic

**DOI:** 10.1155/2015/619734

**Published:** 2015-04-28

**Authors:** Karissa L. Canning, Ruth E. Brown, Sean Wharton, Arya M. Sharma, Jennifer L. Kuk

**Affiliations:** ^1^School of Kinesiology & Health Science, York University, Toronto, ON, Canada M3J 1P3; ^2^The Wharton Medical Clinic, Hamilton, ON, Canada L8L 5G8; ^3^Department of Medicine, University of Alberta, Edmonton, Canada T6G 2G3

## Abstract

*Objectives*. To determine the distribution of EOSS stages and differences in weight loss achieved according to EOSS stage, in patients attending a referral-based publically funded multisite weight management clinic. *Subjects/Methods*. 5,787 obese patients were categorized using EOSS staging using metabolic risk factors, medication use, and severity of doctor diagnosis of obesity-related physiological, functional, and psychological comorbidities from electronic patient files. *Results*. The prevalence of EOSS stages 0 (no risk factors or comorbidities), 1 (mild conditions), 2 (moderate conditions), and 3 (severe conditions) was 1.7%, 10.4%, 84.0%, and 3.9%, respectively. Prehypertension (63%), hypertension (76%), and knee replacement (33%) were the most common obesity-related comorbidities for stages 1, 2, and 3, respectively. In the models including age, sex, initial BMI, EOSS stage, and treatment time, lower EOSS stage and longer treatment times were independently associated with greater absolute (kg) and percentage of weight loss relative to initial body weight (*P* < 0.05). *Conclusions*. Patients attending this publicly funded, referral-based weight management clinic were more likely to be classified in the higher stages of EOSS. Patients in higher EOSS stages required longer treatment times to achieve similar weight outcomes as those in lower EOSS stages.

## 1. Introduction

Common classifications of obesity are based on simple clinical measures such as body mass index (BMI) or waist circumference [1]. However, excess body weight alone does not necessarily translate to poor health outcomes [2]. Individuals with the same BMI can have very different health conditions as many factors other than body weight contribute to disease [3, 4]. Therefore a one size all approach is not sufficient and a more individualized and targeted approach is needed to improve the health with those with the most severe health conditions to ensure they receive the most intense obesity treatment. The Edmonton Obesity Staging System (EOSS) is a five-stage system of obesity classification that considers the metabolic, physical, and psychological parameters in order to determine the optimal obesity treatment [1]. EOSS has been reported to be a better predictor of mortality than BMI or metabolic syndrome [2, 5].

EOSS suggests that only patients in the more severe stages should be recommended weight loss as the health benefits of weight loss in the lower stages of EOSS are unclear [6, 7]. In fact, some research suggests [6, 7] that the ability to lose weight in these “healthy” patients may even be impaired by their healthy metabolic profile. Specifically, “healthy” patients who possess high insulin sensitivity may be more prone to weight gain and have more difficulty losing weight [6, 7]. Additional factors including psychological disturbances [8] such as depression [9] or anxiety disorders [10] may influence weight loss in obese populations through either medication use or emotional overeating which are commonly associated with these conditions [11, 12]. Similarly, physical limitations or chronic pain that may limit the individual from being able to exercise [13, 14] may also hinder the weight management success in these patients.

Therefore the objective of this study was to examine the prevalence of EOSS staging in patients seeking weight management at a publically funded multisite weight management clinic and to determine if there are differences in achieved weight loss using a standard lifestyle treatment or other patient factors within the different stages of EOSS.

## 2. Subjects and Methods

### 2.1. Study Population

Participants were recruited from a publicly funded medically supervised weight management clinic with multiple locations in Ontario, Canada, between July 2008 and December 2014 (*n* = 11,284). The clinic is a multidisciplinary, referral-based clinic for overweight, obese, and diabetic patients who are trying to lose weight and improve health. The main goal of the clinic is to educate patients about weight management and deal with obesity-related chronic conditions. The clinic operates under the Canadian Clinical Practice Guidelines and the National Institutes of Health guidelines for the management of obesity [15, 16] that recommend weight loss for all patients with obesity. EOSS staging was not currently used to determine weight management goals at this clinic.

The clinic is led by a team composed of internal medicine specialists and bariatric educators and all services are provided to the patient at no charge. Participants were excluded from the analyses if they had missing information for age (*n* = 107) or BMI (*n* = 25) or only attended the clinic for one visit and had no weight loss or EOSS data (*n* = 4,097) or had a BMI < 30 kg/m^2^ (*n* = 536), which left a final sample of 5,787 obese patients. Patients who were excluded were similar in age (45.9 ± 13 years) and initial BMI (39.1 ± 7.6) and had a similar proportion of males (22%), as the patients who were included in the final sample. All patients provided written informed consent and all procedures were approved and conducted in accordance with the ethical guidelines of the York University Institutional Review Board.

### 2.2. Weight Management Program

At the initial visit, patients completed a medical questionnaire, had their weight measured using MedWeigh MS-2510 Digital High Capacity Platform Scales (Itin Scale Co, Inc., NY) to the nearest 0.1 kg, had their height measured using a wall-mounted tape measure (McArthur Medical Sales, Inc., ON) to the nearest 0.1 cm, and had their waist circumference, hip circumference, and blood pressure assessed by medical staff. Patients then attended a group information session outlining basic program information and individual meetings with a bariatric educator and physician.

On the second visit, patients received a calorie restricted meal plan aimed at a 500–1000 kcal deficit below calculated daily requirements. Resting Metabolic Rate (RMR) was calculated using the equation by Mifflin et al. [17] and confirmed using indirect calorimetry. Patient weights were assessed at each visit to track weight changes [15]. Patients were encouraged to visit the clinic for weekly weigh-ins and were also expected to attend lifestyle-intervention and educational workshops presented by physicians, exercise specialists, dieticians, and behavioural specialists [15].

### 2.3. Edmonton Obesity Staging System (EOSS)

All information for metabolic risk factors, medication usage, and self-reported doctor diagnosis of obesity-related morbidities was extracted from electronic patient files. We categorized EOSS staging by using the highest-stage risk factor for each patient based on modified operational definitions adapted from Sharma and Kushner [1] displayed in Table 1. For example, a patient with obesity-related subclinical risk factors (borderline high glucose, borderline high blood pressure, etc.), mild physical symptoms, mild psychopathology, and mild functional limitations (stage 1) but diagnosed with arthritis (stage 2) would be categorized as EOSS stage 2. There were no stage 4 patients treated at the clinic.

### 2.4. Weight History

Information on previous weight history was collected in a subsample of patients (*n* = 1479). This included information on self-reported duration of obesity, the age when patients became overweight, and the number of times patients lost weight of 10 lbs or more.

### 2.5. Statistical Analyses

Continuous variables are reported as mean and standard deviations and categorical variables are presented as frequencies and prevalence values. Differences in patient characteristics by EOSS stage were assessed using analysis of variance (ANOVA) with LSD post hoc comparisons for the continuous variables and chi-square tests for the categorical variables. General linear models were used to assess the independent effects of EOSS stage and treatment time on total weight loss (kg) and percentage of body weight loss, adjusting for age, sex, and initial BMI. All statistical analyses were performed using SAS v9.4. Statistical significance was set at alpha < 0.05.

## 3. Results

Patient characteristics stratified by EOSS stage are presented in Table 2. The prevalence of EOSS stages 0, 1, 2, and 3 was 1.7%, 10.4%, 84.0%, and 3.9%, respectively. Patients in EOSS stage 0 were younger and had a lower initial body weight and BMI compared to patients in the higher EOSS stages (*P* < 0.05). Patients in EOSS stages 2 and 3 attended the clinic longer than patients in stage 0 or 1 (*P* < 0.05) (Table 2).

**Figure 1:**
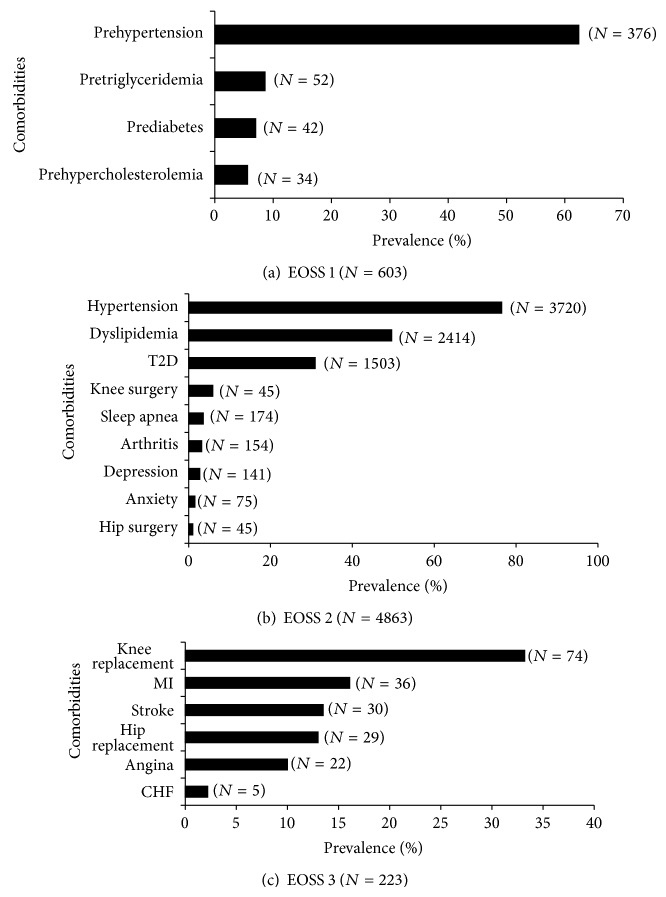
Prevalent comorbidities for patients in EOSS stage 1 (a), stage 2 (b), and stage 3 (c). CHF = congestive heart failure; EOSS = Edmonton Obesity Staging System; MI = myocardial infarction; T2D = type 2 diabetes.

The most common obesity-related comorbidity for patients in EOSS stages 1 and 2 was high blood pressure (62% of stage 1 and 76% of stage 2) (Figures 1(a) and 1(b)). Knee replacement (33%), followed by heart attack (16%), and stroke (13%) were the most prevalent obesity-related comorbidities, in EOSS stage 3 (Figure 1(c)).

**Figure 2:**
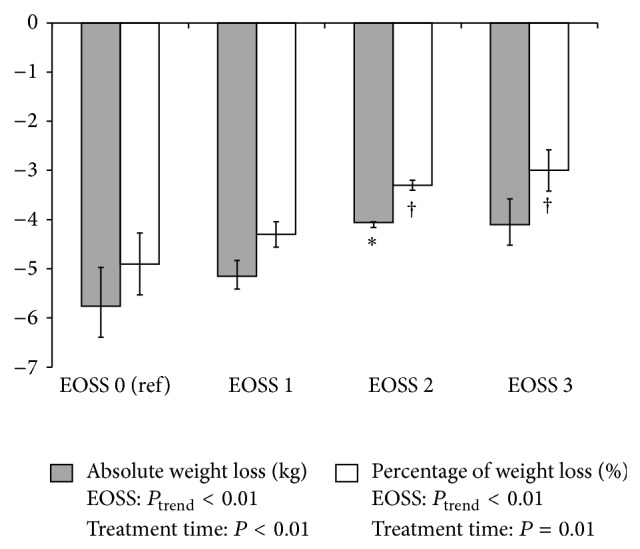
Achieved absolute (kg) and percentage of weight loss relative to initial body weight in patients attending a publicly funded multisite weight management clinic among EOSS stages 0 to 3. Treatment time is in months. EOSS = Edmonton Obesity Staging System; Ref = referent group. Analyses were adjusted for age, sex, initial BMI, and treatment time. ^∗^Significantly different from EOSS stage 0 for absolute weight loss (*P* < 0.05). ^†^Significantly different from EOSS stage 0 for percentage of weight loss (*P* < 0.05).

In the multivariable analyses, attending the clinic for a longer treatment time (*P* < 0.001) was positively related to losing more weight after adjusting for age, sex, and initial BMI. Further, after adjustment for differences in treatment time, being in a lower EOSS stage was associated with greater weight loss (*P* < 0.01 for absolute kg weight loss; *P* < 0.01 for percentage of body weight loss) (Figure 2).

In the subsample of patients with weight history data (*n* = 1479), there was no difference in the age when patients became overweight or the frequency that patients had lost 10 lbs or more by EOSS stage (*P* > 0.05).

## 4. Discussion

We are the first to determine that both treatment time and EOSS stage are important factors predicting weight loss outcomes in a community weight management program. Patients in higher EOSS stages were able to achieve similar weight loss outcomes to patients in lower EOSS stages but may require a longer treatment time if treatment is not tailored for EOSS staging.

Commonly used classification systems of obesity are based on simple clinical measures such as weight, waist circumference [16], or BMI [17, 18]. Currently, weight guidelines suggest that all patients with a BMI > 30 kg/m^2^ should lose weight. However, BMI does not necessarily discriminate differences in health [19], which should be taken into account when making treatment decisions. EOSS uses the patients' physiological, psychological, and physical status to determine the best weight management for that individual [5]. The weight management strategy should reflect the EOSS stage and specific characteristics of the individual. The management for patients in EOSS stages 0 and 1 involves lifestyle interventions including diet and exercise to prevent further weight gain and patients' risk factors and overall health status are monitored [1]. The management for patients in EOSS stages 2 and 3 includes initiating different obesity treatments involving behavioural, pharmacological, and surgical treatment options in addition to close monitoring and management of comorbidities [1]. Previous research suggests that EOSS may aid physicians in identifying obese patients who are at increased mortality risk and in the greatest need of weight management and/or treatment [2, 5]. However, no study has evaluated the distribution of EOSS staging in patients attending clinical weight management.

We demonstrate that there was a higher proportion of patients attending the clinic in EOSS stage 2 than is seen in the general population (EOSS 2: 86% versus 63%) [5]. The higher prevalence of stage 2 patients within the clinic may be positive as these patients have a greater potential to improve or reverse their obesity-related conditions with the proper medical intervention as compared to those patients in lower stages [20]. This difference may be that family physicians or specialists may be more likely to refer overweight and obese patients who have obesity-related comorbidities [21, 22] to a clinical obesity management program. However, under the EOSS treatment model, individuals in stages 0 and 1 are recommended prevention of weight gain, as opposed to weight loss, unlike current guidelines that recommend weight loss for all obese patients. This difference would likely conflict with patient's goals or expectations, and the clinical implications of this change if EOSS was implemented are unclear.

Although the current weight management guidelines [16, 23] recommend weight loss for all obese adults to improve health, weight loss may not be necessary for all obese patients and the ability to lose weight may in fact be impaired in some. “Metabolically healthy” obese patients [24–26] display high levels of insulin sensitivity and favorable blood lipid profiles and are normotensive [27–31]. A high level of insulin sensitivity has been reported to impair weight loss and is associated with weight gain in these patients [6, 7]. We demonstrate that, on average, patients from all EOSS stages were able to attain a similar weight loss. In fact, patients in EOSS stages 0 and 1 lost weight more quickly than those in the upper stages. Currently, under EOSS model, patients in stages 0 and 1 are not recommended weight loss as the long-term health benefits of weight loss are unclear. In this study, it is not known if health outcomes in patients in lower stages improved following weight loss. It is possible that weight loss in these EOSS stages 0 and 1 patients may prevent or delay the transition to higher EOSS stages, as we previously report that those in higher EOSS stages have reported more lifetime weight loss [2]. Thus, longer term studies to test the effect of weight loss in EOSS stages 0 and 1 are needed.

Under the EOSS framework, patients in the upper stages are recommended to lose weight for health. However, in order to achieve similar weight loss outcomes, patients in the upper EOSS stages had treatment times that were over 3 months longer than those in the lower EOSS stages. Patients in the upper EOSS stages may also have factors that may negatively affect weight loss success such as physical limitations or psychological factors. Musculoskeletal disabilities associated with obesity in addition to chronic pain [13, 14] may prevent patients with physical limitations from participating in physical activity which is an important component of weight management. Psychological issues that are commonly associated with obesity such as anxiety disorders and depression may also contribute to the difficulty in losing weight [9]. Commonly used medications to treat severe mental health illnesses or type 2 diabetes are associated with weight gain and increases in appetite [11, 32]. Similarly, stress and anxiety can result in emotional overeating [12] and the production of the stress hormone cortisol [33], which in turn may have negative effects on weight loss ability. These findings reinforce the importance of considering EOSS stage and promoting longer treatment models and weight management goals in clinical weight management.

Limitations and strengths of the current study warrant mention. It is unclear if the results from this study can be generalizable to other weight management clinics, especially since this is a referral weight management clinic that provides government covered services. Due to the flexibility in the EOSS operational criteria, different clinicians or clinics could have different focuses or assessments or patient needs that may alter the pattern of EOSS staging diagnosis. Also, given that EOSS considers a wide range of mental, physical, and functional health impacts, it is unclear to what degree patients within the various EOSS stages may differ in their response to treatment. Also, we were limited by data available to assess all of the comorbidities included in each EOSS stage, which is akin to actual clinical care wherein not every patient receives the same clinical assessments, nor would you expect that every assessment for every potential condition is performed. We were also unable to assess the EOSS weight management model as all patients received similar weight management treatment.

## 5. Conclusions

In summary, patients attending this publicly funded, referral-based weight management clinic were more likely to be in the higher stages of EOSS. This is in line with EOSS recommendations that these patients require more rigorous obesity management and may benefit most from attending a medically supervised weight management clinic. However, these patients may require a longer treatment time in order to attain similar weight losses to patients in lower EOSS stages. Additional studies evaluating the long-term weight and health outcomes across EOSS stages are warranted.

## Figures and Tables

**Table 1:** Edmonton Obesity Staging System (EOSS) study operational definitions.

EOSS stage	Conceptual EOSS definition (Sharma and Kushner, 2009) [1]	Study operational definition
0	No apparent obesity-related risk factors, physical symptoms, psychopathology, functional limitations, and/or impairments of well-being	No EOSS factors reported

1	Presence of obesity-related subclinical risk factors, mild physical symptoms, mild psychopathology, mild functional limitations, and/or impairment of well-being	Any of the following: (i) Glucose ≥ 5.6 mmol/L (ii) Cholesterol ≥ 5.2 mmol/L (iii) Triglycerides ≥ 1.7 mmol/L (iv) HDL ≤ 1.6 mmol/L (v) LDL ≥ 3.3 mmol/L (vi) SBP ≥ 130 mmHg (vii) DBP ≥ 85 mmHg

2	Presence of established obesity-related chronic disease, moderate limitations in activities of daily living, and/or well-being	Any of the following: (i) Glucose ≥ 6.9 mmol/L (ii) Diagnosed type 2 diabetes or type 2 diabetes medication (iii) Cholesterol ≥ 6.2 mmol/L (iv) Diagnosed hypercholesterolaemia (v) Triglycerides ≥ 2.2 mmol/L (vi) HDL ≤ 1.0 mmol/L (vii) LDL ≥ 4.1 mmol/L (viii) Diagnosed hyperlipidaemia or hyperlipidaemia medication (ix) SBP ≥ 140 mmHg (x) DBP ≥ 90 mmHg (xi) Diagnosed hypertension or hypertension medication (xii) Sleep apnea (xiii) Gout (xiv) Arthritis (xv) Anxiety (xvi) Atherosclerosis (xvii) Fatty liver (xviii) Congestive heart failure medication (xix) Blood thinner medication (xx) Depression

3	Established end-organ damage, significant psychopathology, significant functional limitations, and/or impairment of well-being	Any of the following: (i) Angina (ii) Heart attack (iii) Heart failure (iv) Thrombosis (v) Coronary artery disease (vi) Coronary obstructive pulmonary disease (vii) Dyspnea (viii) Exercise dyspnea (ix) Coronary artery bypass surgery (x) Stroke

4	Severe (potentially end-stage) disabilities from obesity-related chronic diseases, disabling psychopathology, functional limitations, and/or impairment of well-being	No data on these factors available to evaluate this stage

HDL: high density lipoprotein; LDL: low density lipoprotein; SBP: systolic blood pressure; DBP: diastolic blood pressure.

**Table 2:** Characteristics of patients attending a publicly funded multisite weight management clinic among EOSS stages 0 to 3.

	EOSS stage 0	EOSS stage 1	EOSS stage 2	EOSS stage 3
*N*	98	603	4863	223
Age (years)	40.5 ± 11.9	44.8 ± 12.5^a^	53.5 ± 12.03^ab^	59.7 ± 11.7^abc^
Sex (% male)	15.3	20.9^a^	29.5^ab^	30.9^ab^
Ethnicity (% White)	77.4	86.7^a^	86.3^a^	88.6^a^
Weight (kg)	104.7 ± 18.5	114.1 ± 23.6^a^	115.8 ± 25.7^a^	112.1 ± 25.3^ac^
BMI (kg/m²)	37.8 ± 5.9	40.8 ± 7.2^a^	41.5 ± 7.7^a^	40.7 ± 8.3^a^
TX time (months)	10.4 ± 11.7	9.4 ± 11.5	12.7 ± 15.0^ab^	14.7 ± 16.0^abc^

EOSS: Edmonton Obesity Staging System; BMI: body mass index; WL: weight loss. ^a^Significantly different from stage 0 (*P* < 0.05). ^b^Statistically significant from stage 1 (*P* < 0.05). ^c^Statistically significant from stage 2 (*P* < 0.05).
